# Increased susceptibility to cortical spreading depression and epileptiform activity in a mouse model for FHM2

**DOI:** 10.1038/s41598-018-35285-8

**Published:** 2018-11-16

**Authors:** Lieke Kros, Karin Lykke-Hartmann, Kamran Khodakhah

**Affiliations:** 1 Dominick P. Purpura Department of Neuroscience, Albert Einstein College of Medicine, 1410 Pelham Parkway South, Bronx, NY 10461 USA; 2 Aarhus University, Department of Biomedicine, Department of Clinical Medicine, Wilhelm Meyers Allé 4, DK-8000 Aarhus C, Denmark; 3 Department of Clinical Genetics, Aarhus University Hospital, Brendstrupgårdsvej 21, DK-8200 Aarhus N, Denmark; 4 Present Address: Department of Neuroscience, Erasmus Medical Center, Wytemaweg 80, 3015 CN Rotterdam, The Netherlands

## Abstract

Migraine is a highly prevalent, debilitating, episodic headache disorder affecting roughly 15% of the population. Familial hemiplegic migraine type 2 (FHM2) is a rare subtype of migraine caused by mutations in the *ATP1A2* gene, encoding the α_2_ isoform of the Na^+^/K^+^-ATPase, predominantly expressed in astrocytes. Differential comorbidities such as epilepsy and psychiatric disorders manifest in patients. Using a mouse model harboring the G301R disease-mutation in the α_2_ isoform, we set to unravel whether α_2_^+/G301R^ mice show an increased susceptibility for epilepsy and cortical spreading depression (CSD). We performed *in vivo* experiments involving cortical application of KCl in awake head-restrained male and female mice of different age groups (adult and aged). Interestingly, α_2_^+/G301R^ mice indeed showed an increased susceptibility to both CSD and epileptiform activity, closely replicating symptoms in FHM2 patients harboring the G301R and other FHM2-causing mutations. Additionally, this epileptiform activity was superimposed on CSDs. The age-related alteration towards CSD indicates the influence of female sex hormones on migraine pathophysiology. Therefore, the FHM2, α_2_^+/G301R^ mouse model can be utilized to broaden our understanding of generalized epilepsy and comorbidity hereof in migraine, and may be utilized toward future selection of possible treatment options for migraine.

## Introduction

Migraine is a highly prevalent, debilitating, episodic headache disorder affecting roughly 15% of the population^1^. A migraine attack is characterized by a severe, long lasting headache that often coincides with nausea and neurological signs and can be preceded by transient neurological symptoms; an aura^2^. Prevalence of migraine is higher in women than men and severity and frequency of headache episodes often decrease after menopause in women or with age in general^1,3^.

A rare subtype of this disorder is familial hemiplegic migraine type 2 (FHM2), an autosomal dominant form of migraine characterized by the occurrence of an aura with hemiparesis (one-sided motor weakness) that shows a high degree of comorbidity with epilepsy^2,4^. In most cases, FHM2 is caused by a loss of function mutation in the *ATP1A2* gene encoding the α_2_-isoform of the Na^+^/K^+^-ATPase, predominantly found in astrocytes^4–6^. The Na^+^/K^+^-ATPase is a pump that actively transports potassium into, and sodium out of, a cell to maintain stable transmembrane ionic gradients^7^. FHM2 related mutations in the *ATP1A2* gene cause haploinsufficiency and result in diminished astrocyte function, rendering them insufficiently capable of clearing excess K^+^ from the extracellular space, and excess glutamate from the synaptic cleft^8,9^.

Two Italian families with several members suffering from FHM2 caused by the G301R mutation have been described^10,11^. This specific mutation causes a particularly severe phenotype with comorbidity of migraine with epilepsy, coma, motor symptoms and psychiatric disorders like depression and obsessive compulsive disorder (OCD)^10,11^. Based on this mutation, a new mouse model for FHM2 was generated and characterized^12^. Initial phenotypical investigation showed hypo-locomotion, OCD-like behavior and stress-induced depression. The phenotypes were generally more severe in females and could be rescued by blocking female sex hormones^12^.

The neurobiological process underlying the migraine aura is thought to be cortical spreading depression (CSD); a wave of initial neuronal and glial depolarization followed by prolonged depression that slowly propagates over the cerebral cortex^13–15^. Although the phenomenon has been described as early as 1944^14^, mechanisms underlying spontaneous initiation and propagation of CSDs, and the relative contribution of glia and neurons, remain incompletely understood^16,17^. Several more recent studies have implicated CSD in causing the subsequent headache through activation of the trigeminal system^13,18–22^. Susceptibility to CSD, defined as a combination of CSD frequency and propagating speed, has been used to determine the validity of previously created FHM1 mouse models^23^. One mouse model harboring the human FHM2-related W887R mutation has been generated^24^ and α_2_^+/W887R^ mice showed a reduced induction threshold for CSD using electrical stimulation in anesthetized animals^25^. Despite several studies, a deep understanding of the neurophysiology associated with CSD and migraine remains to be achieved in experiments in an awake setting to be able to verify and confirm observations from *in vitro* studies and anesthetized settings as well as to allow for assessment of epilepsy susceptibility.

Here, we set to unravel whether a mouse model of FHM2 harboring the G301R mutation (α_2_^+/G301R^ mice) shows an increased susceptibility to CSD and/or a decreased threshold for epileptiform activity. To address this, we performed experiments involving occipital application of KCl in awake, head-restrained heterozygous α_2_^+/G301R^ mice and wild type littermates to determine differences in CSD susceptibility. Interestingly, in addition to generating CSDs, these experiments consistently resulted in epileptiform activity in α_2_^+/G301R^ mice. About half of the α_2_^+/G301R^ mice showed continuous epileptiform activity that was superimposed on CSDs.

## Results

### α_2_^+/G301R^ mice showed an increased susceptibility to CSD

**Figure 1:**
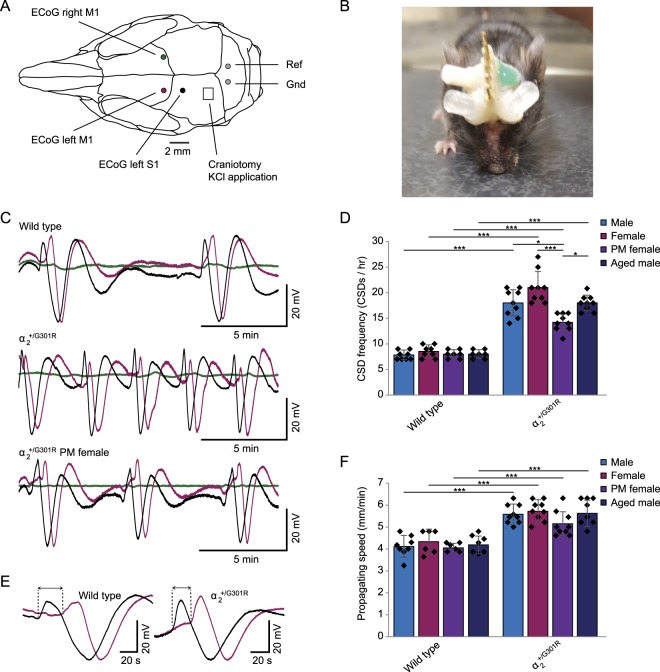
Increased susceptibility to cortical spreading depression in α_2_^+/G301R^ mice. (**A**) Schematic representation of the placement of ECoG electrodes and the craniotomy allowing for the application of KCl. (**B**) Picture of a mouse with implanted electrodes, corresponding connector and small 3D printed cylinders to allow head-restraining secured to its skull. The green area is non-toxic silicone to secure and protect the craniotomy. (**C**) Representative examples of 15 min ECoG traces of M1 left (pink), S1 left (black) and M1 right (green, contralateral) during occipital application of KCl in wild type mice (top), α_2_^+/G301R^ mice (middle) and α_2_^+/G301R^ post-menopausal (PM) females (bottom). (**D**) Quantification of CSD frequency based on 1 hour recordings in all groups of animals; male (light blue), female (pink), post-menopausal (PM) female (purple) and aged male (dark blue) α_2_^+/G301R^ mice and their wild type littermates (N = 7–9). Each black dot represents an animal, error bars represent standard deviations. *p < 0.05, ***p < 0.001 (Mann Whitney *U* tests and Kruskal-Wallis test followed by Dunn’s Multiple Comparison tests with Bonferroni correction; see Table 1) (**E**) Representative example of a single CSD in a wild type (left) and α_2_^+/G301R^ mouse (right) in the time difference in CSD onset between S1 (black) and M1 (pink) is depicted with dotted lines and arrows. (**F**) Quantification of propagating speed for all groups as in (**D**). Error bars represent standard deviations, ***p < 0.001 (Mann Whitney *U* tests and Kruskal-Wallis test and subsequent Dunn’s Multiple Comparison tests with Bonferroni correction; see Table 1).

We first investigated the susceptibility of α_2_^+/G301R^ mice to cortical spreading depression defined by CSD frequency and propagating speed upon occipital application of 300 mM KCl (as described in^23^). Electrocorticographical (ECoG) recordings were made bilaterally from M1 and unilaterally from S1 (left side) while a small craniotomy over the left occipital lobe allowed for application of KCl (Fig. 1A,B). After several days of post-surgical recovery, experiments were performed in awake, head-restrained heterozygous α_2_^+/G301R^ mice (N = 9 per group) and their wild type littermates (N = 8 for males and females and N = 7 for aged males and post-menopausal females). Since migraine is more prevalent in women than men^1^, and often improves after menopause^3^ or with age in general^26^, we compared both genders in two different age groups (2–8 months and 14–20 months).

The occurrence of CSDs upon application of KCl could be observed in the ipsilateral hemisphere of all mice whereas the phenomenon was absent on the contralateral side (Fig. 1C). We found that all groups of α_2_^+/G301R^ mice showed an increased CSD frequency as compared to their wild type littermates (*p* < 0.05 for all; Mann Whitney *U* tests; Fig. 1C,D; Table 1). Whereas no differences were found between wild type groups (*H*(3) = 2.08, *p* = 0.556; Kruskal-Wallis test), we did find significant differences between the groups of α_2_^+/G301R^ mice (*H*(3) = 19.91, *p* < 0.001; Kruskal-Wallis test). More specifically, the post-menopausal females showed a significantly reduced CSD frequency as compared to males (*t*(16) = 13.22, *p* < 0.05), females (*t*(16) = 21.72, *p* < 0.001) and aged males (*t*(16) = 13.28, *p* < 0.05) (Dunn’s Multiple Comparison tests with Bonferroni correction; Fig. 1C,D; Table 1).

**Table 1:** Details of statistical tests performed on data shown in Figure 1D and F.

Compared groups	*N*	*p*-value	*H*, *t* or *U-*value	Statistical test
***Differences in CSD susceptibility***				
Wild types	30	0.556	*H*(3) = 2.08	Independent samples
(Males, females, aged males and PM females)	(8, 8, 7, 7)			Kruskal-Wallis test
α_2_^+/G301R^ mice	36	**<0**.**001**	*H*(3) = 19.91	Independent samples
(Males, females, aged males and PM females)	(9, 9, 9, 9)			Kruskal-Wallis test
α_2_^+/G301R^ males	9	0.507	*t*(16) = −8.50	Dunn’s Multiple Comparison Test
α_2_^+/G301R^ females	9			(Bonferroni correction)
α_2_^+/G301R^ males	9	1.000	*t*(16) = −0.06	Dunn’s Multiple Comparison Test
α_2_^+/G301R^ aged males	9			(Bonferroni correction)
α_2_^+/G301R^ males	9	**<0**.**05**	*t*(16) = 13.22	Dunn’s Multiple Comparison Test
α_2_^+/G301R^ PM females	9			(Bonferroni correction)
α_2_^+/G301R^ females	9	0.519	*t*(16) = 8.44	Dunn’s Multiple Comparison Test
α_2_^+/G301R^ aged males	9			(Bonferroni correction)
α_2_^+/G301R^ females	9	**<0**.**001**	*t*(16) = 21.72	Dunn’s Multiple Comparison Test
α_2_^+/G301R^ PM females	9			(Bonferroni correction)
α_2_^+/G301R^ aged males	9	**<0**.**05**	*t*(16) = 13.28	Dunn’s Multiple Comparison Test
α_2_^+/G301R^ PM females	9			(Bonferroni correction)
Wild type males	8	**<0**.**001**	*U*(15) = 72.00	Mann-Whitney *U* test
α_2_^+/G301R^ males	9			
Wild type females	8	**<0**.**001**	*U*(15) = 72.00	Mann-Whitney *U* test
α_2_^+/G301R^ females	9			
Wild type aged males	7	**<0**.**001**	*U*(14) = 63.00	Mann-Whitney *U* test
α_2_^+/G301R^ aged males	9			
Wild type PM females	7	**<0**.**001**	*U*(14) = 63.00	Mann-Whitney *U* test
α_2_^+/G301R^ PM females	9			
***Differences in Propagating speed***				
Wild types	27	0.890	*H*(3) = 0.63	Independent samples
(Males, females, aged males and PM females)	(8, 6, 7, 6)			Kruskal-Wallis test
α_2_^+/G301R^ mice	36	0.140	*H*(3) = 5.48	Independent samples
(Males, females, aged males and PM females)	(9, 9, 9, 9)			Kruskal-Wallis test
Wild type males	8	**<0**.**001**	*U*(15) = 72.00	Mann-Whitney *U* test
α_2_^+/G301R^ males	9			
Wild type females	6	**<0**.**001**	*U*(13) = 52.50	Mann-Whitney *U* test
α_2_^+/G301R^ females	9			
Wild type males	7	**≤0**.**001**	*U*(14) = 62.50	Mann-Whitney *U* test
α_2_^+/G301R^ aged males	9			
Wild type males	6	**<0**.**001**	*U*(13) = 54.00	Mann-Whitney *U* test
α_2_^+/G301R^ PM females	9			

For propagating speed, calculated by dividing the distance between the two electrodes on the left hemisphere (2 mm) by the time difference in CSD onset, we found a similar pattern of differences between α_2_^+/G301R^ mice and their wild type littermates. All groups of α_2_^+/G301R^ mice showed an increased CSD propagating speed compared to their wild type counterparts (*p* < 0.05 for all; Mann Whitney *U* tests; Fig. 1E,F; Table 1). However, whereas there were clear differences between α_2_^+/G301R^ post-menopausal females and the other α_2_^+/G301R^ groups in CSD frequency, such an effect could not be found for propagating speed between α_2_^+/G301R^ groups (*H*(3) = 5.48, *p* = 0.140; Kruskal-Wallis test) or wild type groups (*H*(3) = 0.63, *p* = 0.890; Kruskal-Wallis test; Fig. 1E,F, Table 1). Together, these data indicate that α_2_^+/G301R^ mice, indeed show a significantly increased susceptibility to cortical spreading depression and remarkably, this susceptibility decreases after menopause in females but not with age in males, suggesting that female hormones influence migraine pathophysiology.

### Increased susceptibility to generalized epileptiform activity in α_2_^+/G301R^ mice

**Figure 2:**
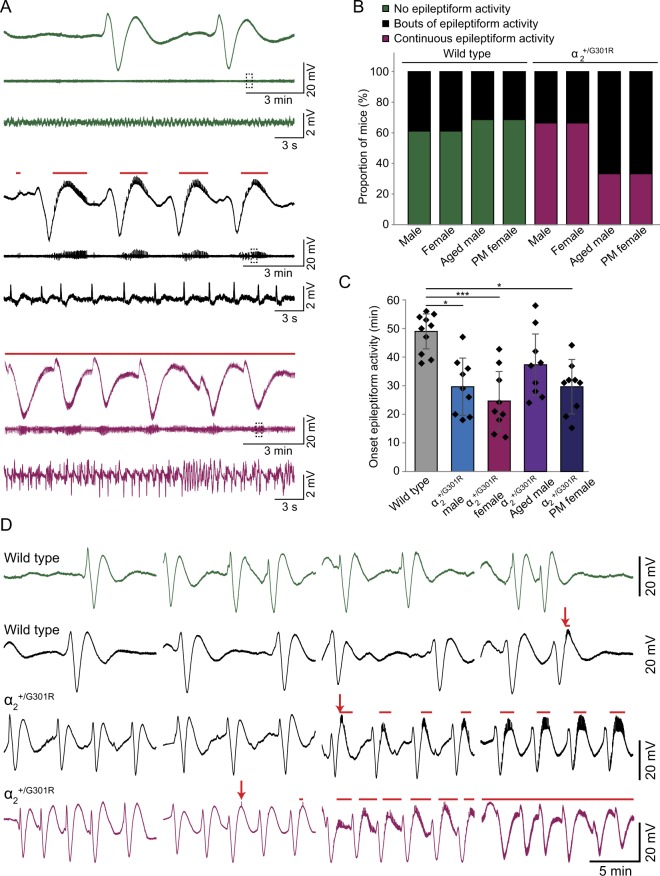
Increased susceptibility to epileptiform activity in α_2_^+/G301R^ mice. (**A**) Representative examples of the last 15 min of 1 hour ECoG recordings (M1) showing various degrees of epileptiform activity. Mice either did not develop epileptiform (green traces), developed bouts of epileptic spikes (black traces) or showed continuous epileptiform activity (pink traces). Traces were filtered using a 0.005 Hz high pass filter (top traces) or a 0.5 Hz high pass filter (middle traces). The bottom traces represent magnifications of the parts of the middle traces depicted by the dashed squares (0.5 Hz high pass filter). The red lines above the black and pink traces indicate the occurrence of epileptiform activity. (**B**) Quantification of the proportion of mice showing either no epileptiform activity (green), bouts of epileptiform activity (black) or continuous epileptiform activity (pink) by the end of the recording (1 hour) for all groups (male, female, aged male and post-menopausal (PM) female α_2_^+/G301R^ mice and their wild type littermates; (N = 7–9)). (**C**) Quantification of onset of epileptiform activity for male, female, aged male and post-menopausal (PM) female α_2_^+/G301R^ mice and wild types (pooled due to low number of animals developing epileptiform activity) (N = 9–10). Error bars represent standard deviations, *p < 0.05, ***p < 0.001 (Kruskal-Wallis test and subsequent Dunn’s Multiple Comparison tests with Bonferroni correction; see Table 2). (**D**) Representative examples of 1 hour ECoG recordings (M1; 0.005 Hz high pass filter) in α_2_^+/G301R^ mice (bottom 2 traces) and wild type littermates (top 2 traces) showing the progression of epileptiform activity over time. Mice either did not develop epileptiform activity (green trace), developed bouts of epileptiform activity (black traces) or progressed to showing continuous epileptiform activity (pink trace). Arrows indicate onset of the epileptiform activity. Like in (**A**), The red lines above the black and pink traces indicate the occurrence of epileptiform activity.

All α_2_^+/G301R^ mice and some wild type littermates showed various degrees of epileptiform activity during CSD experiments (Fig. 2A). This epileptiform activity could range from a few bouts of spikes to a full tonic-clonic seizure- like episode with the accompanying electrophysiological and behavioral correlates (tonic-clonic movements, foam around the mouth and unresponsiveness).

Whereas the majority of wild type littermates did not show any epileptic spikes and the ones that did merely showed some bouts late in the recording, all α_2_^+/G301R^ mice showed epileptiform activity (Fig. 2B). Aged males and post-menopausal females showed a lower percentage of animals that developed continuous epileptiform activity than did younger mice in both the α_2_^+/G301R^ and wild type populations (Fig. 2B). This indicates that susceptibility to epileptiform activity may decrease with age. The onset of the epileptiform activity, defined by the time point at which the first occurrence of epileptic spikes was observed, differed between the groups of α_2_^+/G301R^ mice and wild types (*H*(4) = 20.16, *p* < 0.001; Kruskal-Wallis test; Fig. 2C,D, Table 2) (wild type data was pooled for this analysis due to the low number of wild types developing any epileptiform activity). Post hoc analyses revealed that the onset was earlier in α_2_^+/G301R^ males (*t*(17) = −19.94, *p* < 0.05), females (*t*(17) = −24.61, *p* ≤ 0.001) and aged males (*t*(17) = −20.17, *p* < 0.05) as compared to wild types, whereas the α_2_^+/G301R^ post-menopausal females did not differ from either the other α_2_^+/G301R^ groups or the wild types indicating that susceptibility to epileptiform activity too may decrease after menopause (Dunn’s Multiple Comparison tests with Bonferroni correction; Fig. 2C; Table 2).

**Table 2:** Details of statistical tests performed on data shown in Figure 2C.

Compared groups	*N*	*p*-value	*H* or *t-*value	Statistical test
*Differences in onset of epileptiform activity*				
Wild types (pooled) and α_2_^+/G301R^ mice	46	**<0**.**001**	*H*(4) = 20.16	Independent samples
(Males, females, aged males and PM females)	(10, 9, 9, 9, 9)			Kruskal-Wallis test
Wild types	10	**<0**.**05**	*t*(17) = −19.94	Dunn’s Multiple Comparison Test
α_2_^+/G301R^ males	9			(Bonferroni correction)
Wild types	10	**≤0**.**001**	*t(*17) = −24.61	Dunn’s Multiple Comparison Test
α_2_^+/G301R^ females	9			(Bonferroni correction)
Wild types	10	**<0**.**05**	*t*(17) = −20.17	Dunn’s Multiple Comparison Test
α_2_^+/G301R^ aged males	9			(Bonferroni correction)
Wild types	10	0.519	*t*(17) = −11.94	Dunn’s Multiple Comparison Test
α_2_^+/G301R^ PM females	9			(Bonferroni correction)
α_2_^+/G301R^ males	9	1.000	*t*(16) = 4.67	Dunn’s Multiple Comparison Test
α_2_^+/G301R^ females	9			(Bonferroni correction)
α_2_^+/G301R^ males	9	1.000	*t*(16) = 0.22	Dunn’s Multiple Comparison Test
α_2_^+/G301R^ aged males	9			(Bonferroni correction)
α_2_^+/G301R^ males	9	1.000	*t*(16) = −8.00	Dunn’s Multiple Comparison Test
α_2_^+/G301R^ PM females	9			(Bonferroni correction)
α_2_^+/G301R^ females	9	1.000	*t*(16) = −4.44	Dunn’s Multiple Comparison Test
α_2_^+/G301R^ aged males	9			(Bonferroni correction)
α_2_^+/G301R^ females	9	0.451	*t*(16) = −12.67	Dunn’s Multiple Comparison Test
α_2_^+/G301R^ PM females	9			(Bonferroni correction)
α_2_^+/G301R^ aged males	9	1.000	*t*(16) = −8.22	Dunn’s Multiple Comparison Test
α_2_^+/G301R^ PM females	9			(Bonferroni correction)

The epileptiform activity always started out as a few spikes just after the depression and would progressively develop into broader bouts, which would become continuous in some animals (Fig. 2D). In four cases, the tonic-clonic episode was lethal and mice died shortly after the experiment despite our efforts to rescue each mouse by anesthetizing them after the experiment to abort the epileptiform activity.

**Figure 3:**
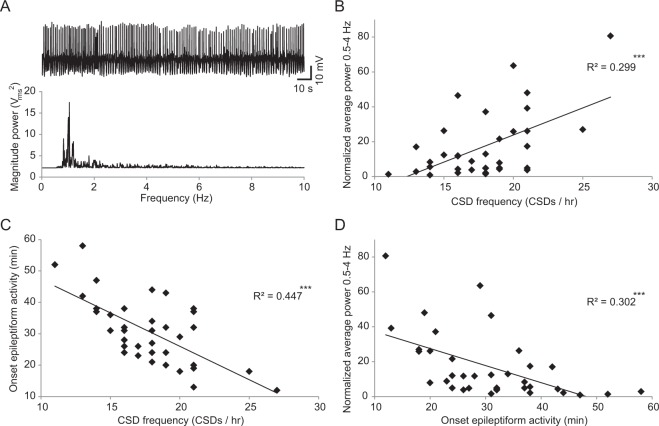
Predictability of onset and severity of epileptiform activity by CSD frequency in α_2_^+/G301R^ mice. (**A**) representative example of severe epileptiform activity (top) and the corresponding power spectrum (bottom). (**B**–**D**) Scatter plots showing linear relations between severity of epileptiform activity (as defined by the normalized power at the dominant frequency of the epileptiform activity) and CSD frequency (**B**), onset of epileptiform activity and CSD frequency (**C**) and severity and onset of epileptiform activity (**D**). ***p < 0.001 (linear regression, see Table 3).

Because both epileptiform activity and CSD can be caused by a high extracellular potassium concentration^13,27,28^ and the efficiency of astrocytes in α_2_^+/G301R^ mice to clear the extracellular space is affected, we next wanted to see whether onset and severity of epileptiform activity could be related to the CSD frequency. Severity of epileptiform activity was defined as the average power at dominant frequency (0.5–4 Hz; Fig. 3A) in the last 10 minutes of the recording normalized to the average power in this frequency range in the first 10 minutes. Combining all data from α_2_^+/G301R^ mice, we a found that a moderate yet significant portion of the variance in both severity of epileptiform activity (*t*(35) = 3.18, *p* ≤ 0.001; linear regression; Fig. 3B, Table 3) and onset of epileptiform activity (*t*(35) = −5.24, *p* < 0.001; linear regression; Fig. 3C, Table 3) could be explained by the variance in CSD frequency; suggesting that a more severe epileptiform phenotype correlates to a more severe degree of CSD susceptibility. A similarly moderate but significant relation was found between onset and severity of epileptiform activity (*t*(35) = −3.84, *p* ≤ 0.001; linear regression; Fig. 3D, Table 3). These analyses suggest that higher susceptibility to CSD and a more severe degree of epileptiform activity indeed often coincide in α_2_^+/G301R^ mice, likely related to increases in extracellular potassium concentration.

**Table 3:** Details of statistical tests performed on data shown in Figure 3B, C and D.

Compared groups	*N*	*p*-value	*t-*value	Statistical test
***Relation CSD susceptibility and epilepsy***				
CSD frequency	36	**≤0**.**001**	*t*(35) = 3.81	Linear regression
**Power at dominant frequency epileptiform activity**				
CSD frequency	36	**<0.001**	*t*(35) = −5.24	Linear regression
**Onset epileptiform activity**				
Onset epileptiform activity	36	**≤0**.**001**	*t*(35) = −3.84	Linear regression
Power at dominant frequency epileptiform activity				

**Figure 4:**
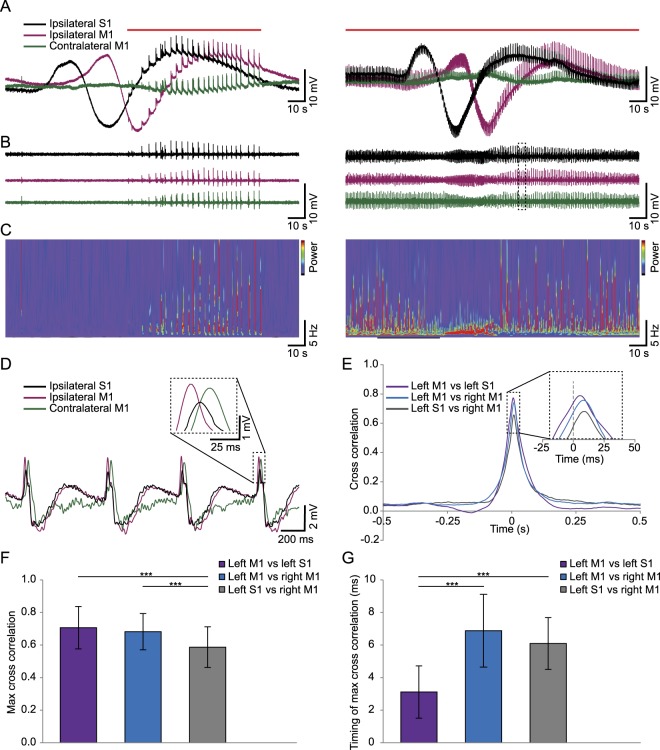
Epileptiform activity is generalized and superimposed on CSDs. (**A,B**) Representative ECoG traces showing bouts of epileptiform activity (left) or late stage continuous epileptiform activity (right) that is generalized (apparent in both hemispheres) and superimposed on the CSD (note that the CSD does not spread to the contralateral hemisphere). All traces are from the same experiment but at 2 different time points; 30 min (left) or 55 min (right). Traces are shown using a 0.005 Hz **(A)** or 0.5 Hz (**B**) high pass filter and a 100 Hz low pass filter (both). The red lines above the traces indicate the occurrence of epileptiform activity. (**C**) Wavelets of the traces shown in (**B**) showing the change in frequency spectra over time. (**D**) Magnification of the part of the trace in (**B**) (right panel) depicted by the dotted square showing the timing of the epileptic spikes in all traces. (**E**) Average cross correlations between all combinations of the three ECoG channels (N = 33 mice). (**F,G**) Quantification of the maximum cross correlations (**F**) and timing of this maximum (**G**) for the three combinations of ECoG channels (M1 left vs S1 left, M1 left vs M1 right and S1 left vs M1 right). Error bars represent standard deviations, ***p < 0.001 (paired *t*-tests; see Table 4).

Further investigation of the spatial and temporal relations between CSD and epileptiform activity, yielded interesting observations. First, whereas a CSD does not spread to the other hemisphere, the epileptiform activity is generalized (Fig. 4A,B). Furthermore, whereas epileptiform activity was confined to the upstroke after the trough in a CSD and the subsequent after-depolarization in early stages of an experiment (Fig. 4A left panel), it became fully superimposed on CSDs in later stages (Fig. 4A right panel) in mice that developed severe epileptiform activity. Although the waveform and power spectra change over the course of a CSD (Fig. 4B,C), epileptic spikes continue during the depression. In order to assess the similarity and timing differences of this epileptiform activity between the different cortical areas, we performed cross-correlations on data obtained from α_2_^+/G301R^ mice. Investigating raw data on a magnified time scale revealed differences in shape and timing between the channels (Fig. 4D) which was reflected in the average cross correlations of the three possible channel combinations (Fig. 4E). Pairwise analyses revealed that the correlation between S1 on the left hemisphere and M1 on the right was significantly lower than the cross correlation between M1 and S1 on the same hemisphere or ipsilateral and contralateral M1 (*t*(32) = 4.30, *p* ≤ 0.001 and *t*(32) = 3.74, *p* ≤ 0.001 respectively; paired *t*-tests; Fig. 4F, Table 4). The latter two combinations of channels were not significantly different (*t*(32) = 0.67, *p* = 0.507). This suggests, as expected, that whereas these correlations were generally quite high (~0.7 on average), epileptiform activity in either two different cortical areas of the same hemisphere or the same cortical area of different hemispheres was better correlated than the activity recorded from S1 and contralateral M1. Analyses of the timing of this peak in the cross-correlogram showed a somewhat different pattern. The epileptiform activity consistently occurs first in the primary motor cortex ipsilateral to the CSD induction site. The time difference between M1 and S1 on the ipsilateral hemisphere is very small and significantly shorter than the time difference between either of those channels and the contralateral M1 electrode (*t*(32) = −8.16, *p* ≤ 0.001 and *t*(32) = −11.09, *p* ≤ 0.001 respectively for M1 and S1; paired *t*-tests; Fig. 4G, Table 4) whereas there is no difference in timing between the ipsilateral electrodes and contralateral M1 (*t*(32) = 1.58, *p* = 0.125). These results indicate that the epileptiform activity occurs first in the ipsilateral hemisphere and likely spreads to the contralateral side from there.

**Table 4:** Details of statistical tests performed on data shown in Figure 4F and G.

Compared groups	*N*	*p*-value	*t-*value	Statistical test
***Max cross correlations epileptiform activity***				
M1 left vs S1 left	33	0.507	*t*(32) = 0.67	Paired *t*-test
M1 left vs M1 right				
M1 left vs S1 left	33	**<0**.**001**	*t*(32) = 4.30	Paired *t*-test
S1 left vs M1 right				
M1 left vs M1 right	33	**≤0**.**001**	*t*(32) = 3.74	Paired *t*-test
S1 left vs M1 right				
***Timing max cross correlations epileptiform activity***				
M1 left vs S1 left	33	**<0**.**001**	*t*(32) = −8.16	Paired *t*-test
M1 left vs M1 right				
M1 left vs S1 left	33	**<0.001**	*t*(32) = −11.09	Paired *t*-test
S1 left vs M1 right				
M1 left vs M1 right	33	0.125	*t*(32) = 1.58	Paired *t*-test
S1 left vs M1 right				

## Discussion

Migraine is a disabling disease which affects a large number of people at different levels. A key characteristic of FHM2 is the appearance of the aura phenomenon prior to the onset of the hemiplegic migraine, associated with CSD. Despite several lines of progress, the mechanisms underlying CSD remain poorly understood. In this study, we show that a mouse model for FHM2 harboring the G301R mutation shows an increased susceptibility to both CSD and generalized epileptiform activity in awake animals. These findings mimic symptoms described in FHM2 patients harboring the G301R mutation in the *ATP1A2* gene^10,11^. Results from *in vivo* recordings in awake, head-restrained mice, show a substantial increase in CSD susceptibility in α_2_^+/G301R^ mice that was similar to data obtained from FHM1 mouse models^23^. Migraine prevalence in humans^1,3^, CSD susceptibility in FHM1 mice^23,29^, and prevalence of psychiatric and motor abnormalities in α_2_^+/G301R^ mice^12^ have been shown to be higher in females and sensitive to blocking the female sex hormone cycle. We therefore assessed CSD susceptibility in adult and aged male and female mice. Whereas FHM1 models show an increased CSD susceptibility in females compared to males^23^, we do not find such an effect of gender. Interestingly, two of nine female α_2_^+/G301R^ mice did show a higher CSD frequency than any of the other α_2_^+/G301R^ mice, suggesting that a gender-based difference may be present in a limited period of the female menstrual cycle. This is in line with our finding that post-menopausal females show a significant decrease in CSD frequency comparable to aged and ovariectomized FHM1 models^23^ and with the rescue of OCD-like behavior, depressed mood and hypo-locomotion in female α_2_^+/G301R^ mice upon suppression of the hormonal cycle^12^. In contrast, aged males did not show a decreased CSD susceptibility, suggesting that the partial rescue of the CSD phenotype in aged females is a consequence of menopause-like hormonal changes rather than age in general. Future pre-clinical studies should be carried out to investigate this phenomenon further, for example by examining whether pharmacological or surgical ablation of sex hormones would also decrease susceptibility to CSD and epileptiform activity in fertile females, and whether this can be reversed by hormone replacement therapy. This could provide a basis for potential clinical trials looking into hormone-based treatment options for women suffering from migraine.

The occurrence of generalized epileptiform activity and coinciding tonic-clonic behavioral manifestations was observed in some wild types and all α_2_^+/G301R^ mice, sometimes with lethal outcome. Interestingly, tonic-clonic seizures have also been described to co-occur with migraine attacks in a family with this mutation^11^. Additionally, the co-occurrence of epileptiform activity and CSD has been shown in rabbits^14,30^ and aneurismal subarachnoid hemorrhage patients^31^. High extracellular potassium concentrations have been known to result in epileptic discharges since the 1950s^27,28,32–35^. In addition, both epilepsy and CSD have also been shown to increase extracellular potassium concentrations^32,36–38^. Given that we are using a KCl solution, and that the resulting CSDs raise potassium levels even further, the occurrence of epileptiform discharges is not unexpected. The progression in the severity of the epileptiform activity suggests that extracellular potassium levels indeed increase over time. The dramatic increase in prevalence and severity of epileptiform activity in α_2_^+/G301R^ mice can be attributed to the impaired functioning of astrocytes resulting in insufficient potassium clearance from the extracellular space^9,39,40^. In combination with a higher CSD frequency, this likely results in higher extracellular potassium levels in α_2_^+/G301R^ mice compared to wild types, resulting in more epileptiform activity. Another potential contributing factor concerns the dependency of the astrocytic glutamate clearance system on α_2_Na^+^/K^+^−ATPase^4,12,41–43^. Both astrocytic glutamate transporters, excitatory amino acid transporter 1 and 2 (EAAT1 and EAAT2) have been shown to be co-localized with α_2_Na^+^/K^+^-ATPase^44^. Glutamate clearance by these transporters depends on the Na^+^ gradient which relies on α_2_Na^+^/K^+^-ATPase^45^. Impairments in astrocytic glutamate uptake from the synaptic cleft have been shown in cultured hippocampal cells from α_2_^G301R/G301R.^ embryonic mice^12^ and in another FHM2 model^39^. Increased extracellular glutamate concentration and EAAT2 deficiency have been shown to cause epilepsy^46,47^, suggesting a defective glutamate system may also contribute to the increased susceptibility in α_2_^+/G301R^ mice. Susceptibility to epileptiform activity has also been shown in the S218L FHM1 mouse model; both spontaneous seizures and epileptic activity post CSD elicitation have been observed, yet such an occurrence has not been mentioned during KCl induced CSDs, likely due to the use of anesthesia^23,29^. Finally, epileptic seizures also occur during or immediately after migraine aura in patients with this mutation^11^. The exclusivity of epilepsy occurrence in relation to aura suggests that it is likely that CSD related changes may underlie the epilepsy phenotype in patients as well. Cross-correlation analyses revealed that this epileptiform activity occurs first in M1 ipsilateral to the CSD induction site. It then occurs in S1 and spreads to the contralateral side, suggesting that the epileptiform activity starts in ipsilateral M1and spreads from there. However, it cannot be excluded that the potassium diffuses into other brain areas, like the hippocampus, and that the epileptiform activity is initiated elsewhere. Yet, regardless of where it is initiated, our data clearly show the occurrence of epileptiform activity during CSD.

In summary, the neuro-electrophysiological *in vivo* evidence for increased susceptibility to CSD and epileptiform activity in the α_2_^+/G301R^ mice suggests that this mouse model closely resembles human symptoms and can be utilized to study the neuropathology underlying migraine disorders. Furthermore, the simultaneous occurrence of epileptiform activity and CSD in awake α_2_^+/G301R^ mice provides a unique opportunity to study the role of neuronal activity in CSD. This could have important implications for the identification of novel therapeutic strategies.

## Materials and Methods

### Animals

All animal experiments were done in accordance with guidelines set, and approved, by IACUC at the Albert Einstein College of Medicine. Data were obtained from 2–8 and 14–20 month old male and female heterozygous α_2_^+/G301R^ mice and their wild type littermates. The older group of female mice were well past the estimated age of 8 months at which murine menopause occurs^48^. Genotyping was done by Transnetyx (Cordova, TN, USA). The presence of the α_2_^+/G301R^ mutation was confirmed by polymerase chain reaction (PCR) using AGCATTTCATCCAGCTGATCACA (forward) and CCCAGGATGAGGGACAGAAC (reverse) primers. The α_2_^+/G301R^ mice^12^ were maintained on a C57BL/6 J background (Jackson laboratory, Bar Harbor, ME, USA). All animals were housed on a reversed light/dark cycle to prevent daytime experiments from interfering with their normal sleep cycle.

### Surgical procedures

Mice were anesthetized using isoflurane (4% in 1.5 L/min O^2^) and kept under anesthesia on a lower dose (1.5–2% in 1.5 L/min O^2^). The head was shaved after which an incision was made to expose the skull. After thoroughly cleaning the exposed surface, a dental adhesive (OptiBond All-In-One; Kerr Corporation, Orange, CA, USA) was applied to the area. Five small burr holes (0.5 mm) were very carefully drilled to allow subdural implantation of three recording electrodes and a reference and ground electrode for electrocorticographical (ECoG) recordings. Recording electrodes were bilaterally placed over the primary motor cortex (M1; ±2 mm ML and +1 mm AP, relative to bregma) and unilaterally over the primary sensory cortex (S1; −2 mm ML (left) and −1 mm AP, relative to bregma). Reference and ground electrodes were placed over the superior colliculi (±1 mm ML and −1 mm AP relative to lambda). A larger craniotomy (~2 mm in diameter) was subsequently drilled over the occipital lobe to permit application of KCl. Dura was left intact during this procedure; if the dura was damaged, the mouse was excluded. Three PFA coated tungsten wires (50 μm) with a small loop at the end to prevent penetration of the dura, were used as recording electrodes and silver chloride balltip electrodes were used as reference and ground electrodes. The electrodes were cautiously positioned in the holes and secured with Charisma (Heraeus Kulzer, Hanau, Germany), a light cured composite. When all electrodes were implanted, the connector was lowered onto the skull and secured with Charisma. A small well was made around the craniotomy to hold the tip of a cotton-tipped applicator during experiments and non-toxic silicone (Kwik-Sil; World Precision Instruments, Sarasota, FL, USA) was subsequently used to close it off. Finally, two small 3D printed, low weight cylinders were secured to the pedestal using Charisma to allow for head-restraining using adjustable magnetic stands (Fig. 1B). After surgery the mouse was given an analgesic (flunixin, 2.5 mg/kg; Bimeda, Dublin, Ireland) and it was kept on a heating pad until fully awake. All animals were given a few days of recovery before experiments started.

### Electrophysiological recordings

ECoG recordings were done in awake head-restrained mice for 1 hour. In order to be able to restrain mice in the setup, they were initially anesthetized shortly (~ 1 min). Recordings were initiated at least 20 minutes after inserting them in the setup to allow for the effects of the anesthesia to wear off completely. After this waiting period, the silicon covering the craniotomy was removed and after visual inspection of the exposed brain (intact dura, healthy looking brain) the experiment was initiated.

In order to assess susceptibility to cortical spreading depression, we performed experiments as described previously^23^. In short: we placed the tip of a small cotton-tipped applicator drenched in KCl solution (300 mM) gently on the dura of the occipital craniotomy to evoke CSDs, and recorded DC ECoG for 1 hour. The cotton-tipped applicator was kept moist throughout the experiment to allow for continuous KCl diffusion. After the hour, the exposed brain was thoroughly flushed with NaCl and the craniotomy was sealed off with silicone again. All mice that showed epileptiform activity during this experiment were anesthetized before being put back in their home cage to stop the pathological activity. Still 4 mice died shortly after a tonic-clonic seizure-like episode. If mice showed extremely epileptiform activity for prolonged periods of time (>20 min), the experiment was aborted prematurely to attempt to prevent death.

All ECoG data was recorded using a custom made amplifier (high pass filter: 0.001 Hz, Low pass filter, 1 kHz, gain: 11), digitized (BNC-2090A; National Instruments, Austin, TX, USA) and stored for further analysis. The extremely high input impedance minimized all potential temporal errors with regard to CSD waveform^49^.

### Data analyses

Custom written algorithms (LabView; National Instruments, Austin, TX, USA) were used for all analyses. All ECoG data was down sampled to 300 Hz and filtered offline (0.005 Hz high pass for CSD data, 0.5 Hz high pass for data involving epileptiform activity, 100 Hz low pass for all). A CSD was identified by the characteristic shifts in DC potential; an initial wave of depolarization followed by pronounced depression. The CSD frequency was determined by counting the number of CSDs per hour and propagating speed was assessed by dividing the distance between the two electrodes (2 mm) by the difference in onset of the wave of depolarization of the first CSD.

Epileptiform activity was identified by the synchronous occurrence of low frequency spikes in the ECoG signal and the coinciding tonic-clonic behavioral correlates. Activity in the contralateral hemisphere was checked in each mouse to assess whether the epileptiform activity was generalized. In each mouse that showed epileptiform activity, it would start in bouts. In a subset of mice, the bouts progressed into continuous epileptiform activity without interruptions. Onset of epileptiform activity was defined as the first moment at which clearly distinguishable spikes occurred in the ECoG. In order to objectively measure the severity of the epileptiform activity, we performed Fast Fourier transforms and calculated the average power at spike frequency (0.5–4 Hz) in the last 10 minutes of a trace and normalized this to the average power at this frequency range in the first 10 minutes. To assess the relation in shape and timing of spikes between channels, cross correlation analyses were performed in α_2_^+/G301R^ mice using a custom written LabView program. We used a Matlab node in LabView and applied the standard xcorr (coeff) function to assess the similarity between two vectors as a function of lag. Three out of the 36 mice were excluded because one of the channels was not working properly.

### Statistical analyses

Due to inequality of variances and because not all variables were normally distributed, statistical differences in CSD frequency, propagating speed and onset of epileptiform activity between the independent groups of mice (young and aged, male and female α_2_^+/G301R^ mice and their wild type littermates) were determined using non-parametric Mann Whitney *U* tests and Kruskal-Wallis tests followed by Dunn’s Multiple Comparison tests with Bonferroni correction in case of a significant result. Predictability of the degree of severity of the epileptiform activity and onset by CSD frequency was tested using linear regression analyses. Differences in maximum cross correlation and timing thereof between the different ECoG channels during epileptiform activity were assessed using paired *t*-tests. Two tailed testing was used in all cases in which a *p*-value was considered significant if below 0.05 (α).

All analyses were performed using SPSS 22 (IBM Corporation, New York, USA).
